# Double-Strand Break Repair Assays Determine Pathway Choice and Structure of Gene Conversion Events in *Drosophila melanogaster*

**DOI:** 10.1534/g3.113.010074

**Published:** 2013-12-24

**Authors:** Anthony T. Do, Joseph T. Brooks, Margot K. Le Neveu, Jeannine R. LaRocque

**Affiliations:** Department of Human Science, Georgetown University Medical Center, Washington, DC 20057

**Keywords:** homologous recombination, nonhomologous end joining, gene conversion, double-strand break repair, *Drosophila*

## Abstract

Double-strand breaks (DSBs) must be accurately and efficiently repaired to maintain genome integrity. Depending on the organism receiving the break, the genomic location of the DSB, and the cell-cycle phase in which it occurs, a DSB can be repaired by homologous recombination (HR), nonhomologous end-joining (NHEJ), or single-strand annealing (SSA). Two novel DSB repair assays were developed to determine the contributions of these repair pathways and to finely resolve repair event structures in *Drosophila melanogaster*. Rad51-dependent homologous recombination is the preferred DSB repair pathway in mitotically dividing cells, and the pathway choice between HR and SSA occurs after end resection and before Rad51-dependent strand invasion. HR events are associated with long gene conversion tracts and are both bidirectional and unidirectional, consistent with repair via the synthesis-dependent strand annealing pathway. Additionally, HR between diverged sequences is suppressed in *Drosophila*, similar to levels reported in human cells. Junction analyses of rare NHEJ events reveal that canonical NHEJ is utilized in this system.

Maintenance of genomic integrity is essential for cell survival and accurate transmission of genetic information. DNA damage is one source of genomic instability, which may arise from exogenous sources or endogenous cellular processes. A particularly deleterious type of DNA damage is the double-strand break (DSB), in which both strands of the DNA molecule are broken. An inability to repair DSBs can lead to cell death, genome rearrangement, and/or cellular transformation (Ferguson and Alt. 2001). DSBs can be repaired by homologous recombination (HR), single-strand annealing (SSA), and nonhomologous end-joining (NHEJ).

In HR, an unbroken homologous sequence is used as a template for repair of the broken sequence. HR is initiated by 5′ to 3′ end resection (Figure 1). Resection is followed by Rad51-dependent strand invasion (McIlwraith *et al.* 2000; Sugawara *et al.* 1995). A homologous sequence is used as a template for repair synthesis, resulting in formation of a D-loop. After D-loop formation, HR can proceed by one of two models. In the canonical DSB repair (DSBR) model proposed by Szostak *et al.* (1983), repair synthesis is followed by formation of a double Holliday junction (Figure 1A). Depending on how the junction is resolved, the resulting product is either a noncrossover or a crossover. The second HR pathway, synthesis-dependent strand annealing (SDSA), involves repair synthesis, dissociation of the newly synthesized strand, and ligation to the other DNA end, resulting in a noncrossover (Figure 1B). Although the DSBR pathway is required for crossover formation during meiosis, SDSA is the predominate pathway for mitotic DSBs in yeast, during gap repair in *Drosophila*, and during mitotic DSB repair in mammalian cells (LaRocque and Jasin 2010; Nassif *et al.* 1994; Paques and Haber 1999; Richardson *et al.* 1999). Recombination intermediates in both DSBR and SDSA contain heteroduplex DNA (hDNA). hDNA is recognized and corrected by mismatch repair machinery and can result in gene conversion, where genetic information is converted to that of the homologous donor template. DSBR in wild-type cells is particularly sensitive to sequence homology, because recombination between diverged sequences is suppressed in *Escherichia coli*, *Saccharomyces cerevisiae*, and mammalian cells (Chen and Jinks-Robertson 1999; Datta *et al.* 1996; Datta *et al.* 1997; Elliott and Jasin 2001; LaRocque and Jasin 2010; Rayssiguier *et al.* 1989; Selva *et al.* 1995; Selva *et al.* 1997). If a DSB occurs between direct repeats, as in repetitive DNA, then strand resection can reveal complementary sequences that anneal, resulting in SSA (Figure 1) and loss of the intervening sequence.

**Figure 1:**
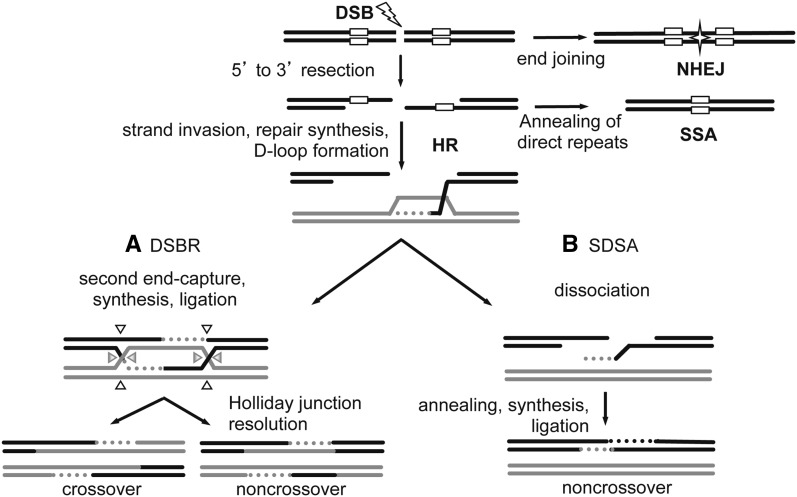
Models of DSB repair. DSBs can be repaired by homologous recombination (HR), single-strand annealing (SSA) or nonhomologous end-joining (NHEJ). In NHEJ, processed ends are joined by ligation (star). HR repair is initiated by 5′ to 3′ resection at the DSB. If the DSB occurs between direct repeats (white boxes), then extensive resection followed by annealing of the direct repeats results in SSA. Otherwise, the resected 3′ overhang invades the homologous template (gray) to initiate repair synthesis (gray dotted line). (A) In the DSBR model, the second strand of the DSB is captured, followed by repair synthesis, and then the newly synthesized strands are ligated to form a double Holliday junction (dHJ). Depending on how the dHJ is cleaved (arrow heads), resolution can result in a crossover or a noncrossover. (B) In SDSA, the newly synthesized strand dissociates, anneals to the other end, the gap is filled in, and nicks are ligated to result in a noncrossover product. The newly synthesized strands in both DSBR and SDSA form heteroduplex DNA (hDNA) between the black and gray sequences. hDNA can be repaired by mismatch repair, resulting in gene conversion (not shown). Direct repeats are shown only for SSA for simplicity.

Another DSB repair pathway, NHEJ, involves end recognition, end processing, and ligation (Lieber *et al.* 2008) (Figure 1). NHEJ can be associated with insertions and deletions that reveal small (<5 nt) microhomologies, resulting in loss or gain of genetic information at the site of the break. Thus, whereas HR is considered error-free, both SSA and NHEJ may result in mutagenic alteration of sequences at the DSB. Despite the potential mutagenic outcome of NHEJ, it is used throughout all phases of the cell cycle and is the predominant pathway in G1 phase of mammalian cells (Rothkamm *et al.* 2003). Considering the various pathways cells use to repair DSBs, pathway choice is determined by several factors including cell-cycle phase, tissue specificity, lesion structure, and organism (Shrivastav *et al.* 2008).

To address the contributions of repair pathways of a simple DSB in the context of a whole organism, we developed the DR-*white* (direct repeat of *white*) assay that detects repair of an inducible simple DSB in *Drosophila melanogaster*. DR-*white* directly measures the frequency of HR and also detects SSA and NHEJ events. An additional novel DSB repair reporter, DR-*white.mu*, determines gene conversion tract length and directionality at high resolution and measures recombination between diverged sequences. We found that Rad-51–dependent HR dominates these DSB repair events, but recombination between diverged sequences is suppressed in *Drosophila*. Additionally, gene conversion events are long, both bidirectional and unidirectional, and the homologous donor sequence remains unchanged. These repair structures suggest a recombination mechanism that includes two-ended strand invasion followed by extensive repair synthesis and gene conversion via SDSA.

## Materials and Methods

### DNA manipulations

DR-*white* was constructed by a multi-step cloning process. *Sce.white* was created by ligating a polylinker into the *Sac*I-digested *white* cDNA. The polylinker contains overhangs complementary to the *Sac*I-induced overhangs, an 18-bp I-*Sce*I recognition sequence, and an additional base pair to create an in-frame premature termination signal (forward, 5′ GTAGGGATAACAGGGTAATAGCT; reverse, 5′ ATTACCCTGTTATCCCTACAGCT). *iwhite* was created by PCR amplification of a truncated fragment of *white* cDNA that omits the 5′ UTR, start codon, the carboxy-terminal 30 amino acids, and the 3′ UTR. The *iwhite* PCR fragment with flanking restriction sites was cloned into *Pst*I/*Not*I–digested pBlueScript.KS^−^.attB vector to create pBSKS^−^.iwhite.attB. DR-*white* was created using a three-way Gateway LR Clonase II reaction of *Sce.white*, 5.1 kb of chromosome *X* DNA (which includes the *y*+ transgene), and *iwhite*.attB (Life Technologies, Grand Island, NY) following the manufacturer’s protocol.

DR-*white.mu* was constructed as DR-*white*, with the incorporation of 28 silent polymorphisms along the length of *iwhite* of pBSKS^−^.*iwhite*.attB using QuikChange Lightning Multi Site-directed Mutagenesis (Agilent Technologies, La Jolla, CA) and confirmed by sequencing. For a list of the mutagenesis primers and location of polymorphisms, see Supporting Information, Table S1.

### *Drosophila* stocks and genetics

*Drosophila* were maintained on standard Nutri-fly Bloomington Formulation medium (Genesee Scientific, San Diego, CA) at 25°. Purified DR-*white* and DR-*white.mu* constructs were injected and integrated at the 51C1 locus of FlyC31 line M{3xP3-RFP′}ZH-51C using PhiC3 integrase system (Bischof *et al.* 2007) (BestGene Inc, Chino Hills, CA). Stable transformants were selected based on *y+* expression and locus integration confirmed by PCR. Five independent lines were established for both DR-*white* and DR-*white.mu*. Four lines of DR-*white* and three lines of DR-*white.mu* were preliminarily tested for DSB repair. One DR-*white* line and one DR-*white.mu* with HR repair frequencies closest to the average HR repair frequency of all lines analyzed were selected and used in all subsequent experiments. The heat-inducible I-*Sce*I transgene was on chromosome *2* (Rong and Golic 2003). The *spn-A* mutants were compound heterozygotes of *spn-A^093A^* (Staeva-Vieira *et al.* 2003) and *mus309^N1^* (McVey *et al.* 2007) *spn-A^057^* (Staeva-Vieira *et al.* 2003).

### DSB repair assay

To induce DSBs, females containing DR-*white* or DR-*white.mu* were crossed to males containing the heat-inducible I-*Sce*I transgene (Wei and Rong 2007). After 3 d, flies were removed and 0- to 3-d-old embryos were heat-shocked in a 38° water bath for 1 hr. Single F1 males containing both DR-*white* (or DR*-white.mu*) and heat-inducible I-*Sce*I transgene were crossed to 4–5 *y w* females in vials. For each experiment, F2 progeny from 14–49 individual male germlines were scored. DSB repair pathways were determined in F2 progeny containing DR-*white* or DR-*white.mu*. For molecular analyses, 1–2 isolates from each vial were analyzed to avoid frequency biases attributable to potential germline jackpot events (Luria and Delbruck. 1943).

### Molecular analyses

Genomic DNA was isolated from single flies as previously described (Gloor *et al.* 1993). *Sce.white* was PCR-amplified using *Sce.white*-specific primers (forward, DR-*white*1.3, 5′ GTTTTGGGTGGGTAAGCAGG; reverse, DR-*white*1a, 5′ AGACCCACGTAGTCCAGC) using SapphireAmp Fast PCR Master Mix (Clonetech, Mountain View, CA). For I-*Sce*I and *Sac*I digests, PCR products were directly digested (New England Biolabs, Ipswitch, MA). For NHEJ junction analyses, *Sce.white* PCR products were directly sequenced across the break site with DR-*white*2 (5′ ATGCAGGCCAGGTGCGCCTATG). For gene conversion tract analyses, DR-*white.mu* HR events were amplified with DR-*white*1.3 and DR-*white*1a, and PCR products were directly sequenced with primers DR-*white*2 and DR-*white*2a (5′ TGGCAACCATCGTTGTCTG) for incorporation of polymorphisms. For donor sequence analyses, *iwhite*.*mu* was amplified using *iwhite*-specific primers (forward, DR-*white*3, 5′ GTATAATAAAGTTGGGCC; reverse, *iwhite*.a, 5′ GCAGATCGGCGGCGGAGAAGTT). Products were directly amplified by nested PCR (forward, DR-*white*2; reverse, DR-*white*2a). Nested PCR products were gel-purified and sequenced with primer DR-*white*2. Chromatograms were analyzed for changes in donor sequence. SSA events were confirmed by amplification across DR-*white* or DR-*white.mu* using DR-*white*1.3 and DR-*white*4a (5′ CGAATTCCTGCAGTTGCAG).

## Results

### DR-*white* measures HR repair frequency and detects NHEJ and SSA repair events of a simple DSB

**Figure 2:**
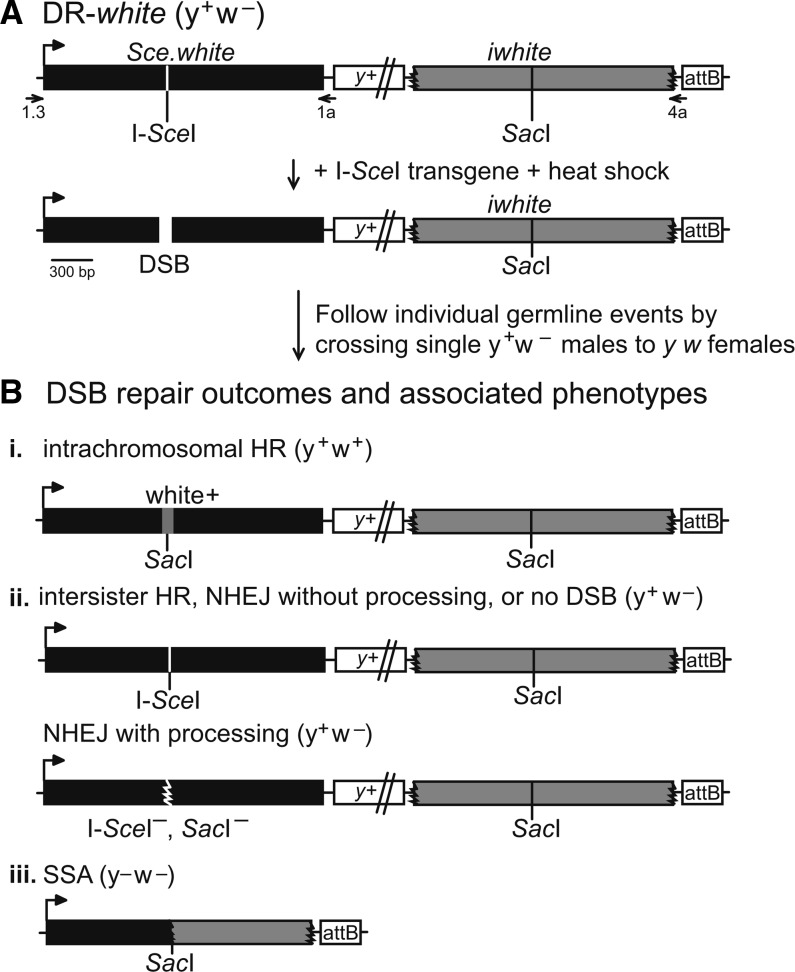
DR-*white* and DR-*white.mu* measure repair of an induced DSB. (A) To analyze repair of an inducible chromosomal DSB, an I-*Sce*I recognition sequence is inserted into the wild-type *Sac*I recognition sequence of *white* cDNA, resulting in a defective *white* sequence (*Sce.white*; black). The second *white* sequence is defective because of 5′ and 3′ truncations (*iwhite*; gray). Integration of DR-*white* is targeted using the attB sequence and followed with the *yellow* (*y*+) transgene (not to scale). Embryos and larvae containing both DR-*white* and a heat-shock–inducible I-*Sce*I transgene are heat-shocked and crossed to *y w* females to score individual germline repair events. (B) After I-*Sce*I cleavage, three phenotypes associated with DSB repair outcomes occur. (i) Noncrossover intrachromosomal HR occurs with gene conversion of the I-*Sce*I sequence to wild-type *Sac*I sequence (conversion shown in gray), resulting in white+ recombinants. (ii) Retention of the y+ w− parental phenotype occurs after intersister HR, NHEJ without processing, no DSB, or NHEJ with processing. The latter can be identified by amplification of *Sce.white* with primers DR-*white*1 and DR-*white*1a (1, 1a), followed by *in vitro* cleavage of the PCR product with both I-*Sce*I and *Sac*I. Junctions of NHEJ with processing events are analyzed by sequencing *Sce.white* PCR products. (iii) SSA results from extensive resection and annealing of direct repeats and loss of intervening *y*+ sequence. These events are confirmed by 2.0-kb amplification across DR-*white* with primers DR-*white*1.3 and DR-*white*4a (1.3, 4a). Phenotypes of the DSB repair events and status of DSB break site sequence are given for all outcomes.

The ability to determine DSB repair pathway choice in multicellular organisms requires a system that can detect repair by HR, NHEJ, and SSA after a single inducible DSB. The novel DR-*white* reporter detects repair of DSBs induced by cleavage of a specific recognition sequence by the rare-cutting homing meganuclease I-*Sce*I. I-*Sce*I cleavage creates a simple chromosomal DSB, which may be more physiologically similar to endogenous DNA breaks that arise during replication than from other exogenous sources (*e.g.*, those generated by exposure to ionizing radiation or double-strand gaps from *P*-element excision). DR-*white* contains two nonfunctional direct repeats of *white*: *Sce.white* and *iwhite* (Figure 2A). *Sce.white* is nonfunctional because of an 18-bp I-*Sce*I recognition sequence and one additional base pair inserted at the wild-type *Sac*I sequence, resulting in an in-frame premature stop codon. The second repeat of *white*, *iwhite*, is nonfunctional because of 5′ and 3′ truncations removing the promoter, 5′ UTR, start codon, the last 30 amino acids, and the 3′ UTR. The two *white* repeats are separated by 5.1 kb of chromosome *X* sequence that includes the *yellow* transgene (*y+*). DR-*white* also contains a 285-bp attB integration sequence downstream of *iwhite*. Inclusion of the attB sequence ensures integration at known attP landing sites within the *Drosophila* genome (Bischof *et al.* 2007). Stable germline transformants were selected based on expression of the *y+* transgene in a *y w* background.

DSBs are introduced by crossing a heat-shock–inducible I-*Sce*I endonuclease transgene into DR-*white* flies and exposing their 0- to 3-d-old embryos and larvae to heat shock (38° for 1 hr). I-*Sce*I cleaves at the I-*Sce*I recognition sequence of *Sce.white* and the DSB is repaired in premeiotic germline cells and mitotically dividing somatic cells. To analyze individual premeiotic germline events, males with both DR-*white* and I-*Sce*I transgene were crossed to *y w* females. Each progeny from this cross represents a single DSB repair event and can be distinguished based on phenotype and/or molecular analyses (Figure 2B). Accurate repair by intrachromosomal noncrossover HR results in restoration of the wild-type *Sac*I site and *w*+ expression (Figure 2B, i). The y+ w− progeny result from three potential outcomes: no DSB formation; repair by intersister HR; or repair by NHEJ (Figure 2B, ii). NHEJ events can be detected from this group by molecular analysis at the I-*Sce*I site. Loss of the I-*Sce*I recognition sequence suggests repair by NHEJ with processing. Retention of the I-*Sce*I site suggests either no DSB or no detectable DSB repair (such as NHEJ without processing or intersister HR). The y− w− progeny results from loss of the *y*+ coding sequence by extensive end resection (>4.4 kbp), followed by end-joining (incomplete HR) or extensive end resection of >7.2 kbp, followed by SSA of the direct repeat sequence (Figure 2B, iii). SSA events can be confirmed by loss of *y*+ transgene after amplification across DR-*white*.

### DSBs are repaired via homologous recombination

**Figure 3:**
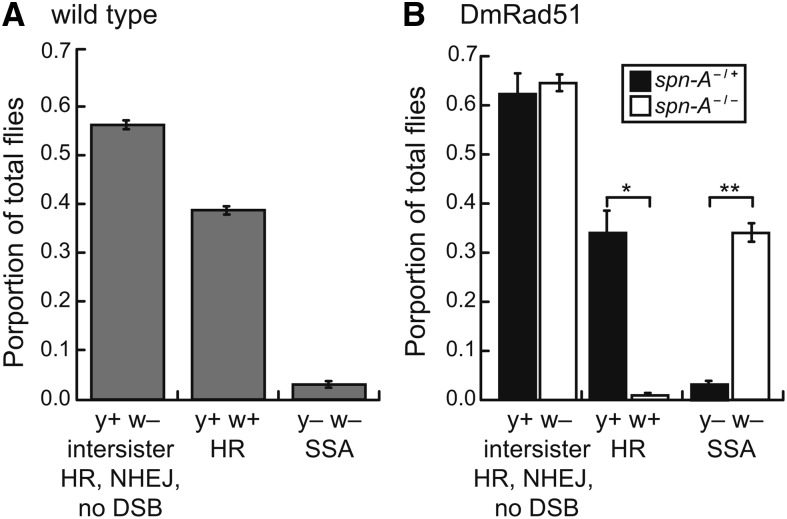
Phenotypic outcomes of DSB repair events. (A) Individual germline DSB repair events were determined based on the y w phenotype in wild-type flies. Results shown are averages and SD of four independent experiments with a total of 5685 flies scored. (B) DSB repair events in a *spn-A* (DmRad51) mutant background. Results shown are averages and SD of three independent experiments, directly comparing each genotype. *P < 0.05 and **P < 0.01 by pairwise Student *t* test. For total number of progeny scored for each experiment, see Table S2.

The DR-*white* reporter was targeted to 51C1 locus of chromosome *2* in wild-type flies and DSB repair was analyzed; 39.7% of progeny from wild-type flies were w+, suggesting DSB repair via homologous recombination (Figure 3A and Table S2). The contribution of non-HR events was also analyzed in wild-type flies; 3.0% of DSB repair events lost the *y*+ transgene, which could occur by either end resection followed by end-joining or end resection followed by SSA. SSA results in loss of the intervening *y+* sequence, which is detectable by amplification of a 2.0-kb fragment. Individual y− w− events were molecularly analyzed to confirm repair by SSA. Of all y− w− analyzed, 90.7% (n = 43) amplified the 2.0-kb SSA product, demonstrating that a majority of y− w− progeny resulted from SSA of the *white* direct repeats as predicted in Figure 2B, iii. As expected, 100% of confirmed SSA events converted the I-*Sce*I to *Sac*I (n = 51).

The remaining 57.3% of progeny from wild-type flies maintained the parental y+ w− phenotype, indicating either DSB repair by NHEJ with end processing, no DSB, or no detectable DSB repair (such as intersister HR or NHEJ without processing). To identify NHEJ with end processing events, y+ w− events were isolated, *Sce.white* PCR-amplified, and PCR products were subjected to *in vitro* digestion by I-*Sce*I and *Sac*I. Only 7.7% of y+ w− flies demonstrated no cleavage by I-*Sce*I or *Sac*I because of loss of the DSB break site by end processing (n = 91) (Table 1). The structures of these rare NHEJ events were further characterized by amplifying and sequencing these I-*Sce*I− and *Sac*I*−* events. The NHEJ events were similar in structure to classical NHEJ with limited processing in that all events had <11 nt deletions/insertions and a subset of these (42.9%) had short microhomologies (n= 7) (Table 2).

**Table 1:** Frequency of nonhomologous end-joining events

**Genotype**	**DSB Repair Assay**	**Experiment No.**	**No. y+ w− Isolates Analyzed***^a^*	**No. NHEJ (%)***^b^*
Wild-type	DR-*white*	1	18	1 (5.6)
		2	18	2 (12.5)
		3	20	0 (0.0)
		4	35	4 (11.4)
		**Total**	**91**	**7 (7.7)**
	DR-*white.mu*	1	19	2 (10.5)
		2	16	0 (0.0)
		3	17	3 (17.6)
		**Total**	**52**	**5 (9.6)**
*spn-A*/+	DR-*white*	1	44	2 (4.5)
		2	30	3 (10.0)
		3	22	3 (13.6)
		**Total**	**88**	**8 (8.3)**
*spn-A/spn-A*	DR-*white*	1	35	4 (11.4)
		2	37	6 (16.2)
		3	25	0 (0.0)
		**Total**	**97**	**10 (10.3)**

a 1 or 2 y+ w– isolates per germline were analyzed.

b Nonhomologous end-joining with processing, as determined by no cleavage of *Sce.white* PCR by I-*Sce*I or *Sac*I.

**Table 2:** Non-homologous end-joining junction sequences

*Sce.white* Sequence			
	GAGCTGTTTGAGCT**G*TAGGGATAA***	***CAGGGTAAT*AGCT**CTTTGACA	
Isolate No.	Junction Sequence		Δ bp
15	GAGCTGTTTGAGCT**G*TAGGGATAA***	***-—————–T*AGCT**CTTTGACA	−8
19	GAGCTGTTTGAGCT**G*TAGGGATAA***	***-—————–T*AGCT**CTTTGACA	−8
21	GAGCTGTTTGAGCT**G*TAGGGATAA***	***-—————–T*AGCT**CTTTGACA	−8
7	GAGCTGTTTGAGCT**G*TAGGGATAA*** (TAA)	***CAGGGTAAT*AGCT**CTTTGACA	+3
13	GAGCTGTTTGAGCT**G*TAGGG*——–-**	***CAGGGTAAT*AGCT**CTTTGACA	−4
22	GAGCTGTTTGAGCT**G*TA-GGATAA***	***CAGGGTAAT*AGCT**CTTTGACA	−1
40	GAGCTGTTTGAGCT**——————––-**	***CAGGGTAAT*AGCT**CTTTGACA	−10
1(mu)	GAGCTGTTTGAGCT**G*TAGGGATAA***	***-—————–T*AGCT**CTTTGACA	−8
11(mu)	GAGCTGTTTGAGCT**G*TAGGG*——–-**	***CAGGGTAAT*AGCT**CTTTGACA	−4
22(mu)	GAGCTGTTTGAGCT**G*TAGGGATAA***	***-—————–T*AGCT**CTTTGACA	−8
25(mu)	GAGCTGTTTGAGCT**G*TAG***(T)***GGATAA***	***CAGGGTAAT*AGCT**CTTTGACA	+1
27(mu)	TTCCCGCTTACACACAATTGCACA*^a^*	***CAGGGTAAT*AGCT**CTTTGACA	−780, +818

Sequence inserted at wild-type *Sac*I site is indicated in bold, creating *Sce.white*. The 18-bp I-*Sce*I recognition sequence is italicized. Sequence of isolated nonhomologous end-joining junction events are shown. Microhomologies are underlined, and changes in bp number are indicated as either deletion (−) or insertion (+).

a 27(mu) included a deletion of 780 bp from *Sce.white* (upstream of DSB) and 818-bp insertion of y+ in the opposite orientation.

To confirm that w+ progeny represented canonical intrachromosomal homologous recombination events, DSB repair was analyzed in flies deficient for DmRad51 (*spn-A*) (Staeva-Vieira *et al.* 2003). Only 0.95% of all repair events were y+ w+ (Figure 3B) (*P* < 0.05), demonstrating that w+ events in the DR-*white* assay result from Rad51-dependent HR. A concurrent increase in y− w− progeny was observed (33.6%). DR-*white* was amplified in y− w− isolates from *spn-A* mutants and 85.8% underwent SSA (n = 148) as evidenced by the loss of the intervening sequence between the *white* direct repeats. SSA-positive PCR products from *spn-A* mutants were digested by *Sac*I and 100% cleaved, indicating conversion of the DSB site to the wild-type *Sac*I recognition sequence (n = 127). The y+ w− isolates were analyzed for NHEJ with processing by amplifying *Sce.white* and digesting PCR products with I-*Sce*I. Of 96 *spn-A/+* events analyzed, 8 (8.3%) did not cleave with I-*Sce*I, and 10 out of 97 (10.3%) events from *spn-A* mutants did not cleave with I-*Sce*I (3 independent experiments; *P* = 0.8 using Fisher exact test). These genetic and molecular analyses demonstrate that a majority of DSB repair events detected in our system are repaired through the Rad51-dependent HR pathway in wild-type flies.

### DR-*white.mu* is a novel assay that determines gene conversion structures and changes to donor sequence

**Figure 4:**
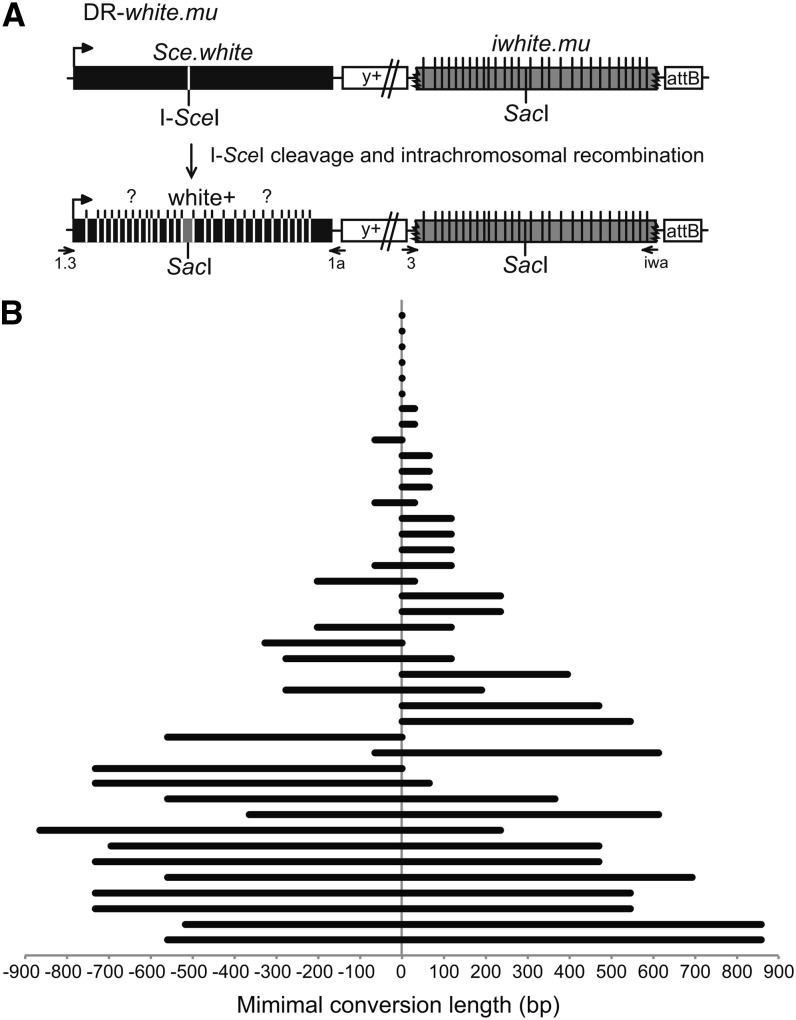
DR-*white.mu* determines gene conversion tract direction and length. (A) DR-*white.mu* is similar to DR-*white* (Figure 2), except it contains 28 silent polymorphisms along the length of the *iwhite* donor sequence (not to scale). For a list of the polymorphisms and exact location, see Table S1. After I-*Sce*I expression and cleavage, homologous recombination using *iwhite.mu* as the donor sequence results in restoration of the wild-type *Sac*I sequence and white+ phenotype. Gene conversion tracts include at least the *Sac*I site (gray) and may or may not include polymorphisms to the left or right of the break (indicated by “?”). To analyze changes to the donor sequence, *iwhite* was amplified with primers DR-*white*3 and *iwhite*.a (3, iwa) and sequenced. (B) To determine gene conversion direction and length, *Sce.white* was amplified from y+ w+ isolates with primers 1.3 and 1a, and then sequenced for conversion to the polymorphisms of the *iwhite.mu* donor sequence. Minimal tract lengths of 41 y+ w+ HR repair events are shown, including the last polymorphism converted. Distance converted to the left and to the right of the *Sac*I site (0) is given.

Our results utilizing DR-*white* suggest that homologous recombination is the preferred DSB repair pathway in *Drosophila*. The structure of gene conversion tracts may give insight to the mechanism by which simple DSBs are repaired by HR in mitotic cells. To determine the structure of gene conversion events, DR-*white.mu* was created (Figure 4A). DR-*white.mu* is identical to DR-*white* but contains 28 silent polymorphisms distributed along the length of the *iwhite* donor sequence (*iwhite.mu*; see Table S1 for exact polymorphisms and locations). DR-*white.mu* was also targeted to the 51C1 locus of chromosome *2* and stable transformants were selected based on *y+* expression. As with the DR-*white* DSB repair assay, repair by HR restores the wild-type *white* sequence (Figure 4A). Individual gene conversion tracts were isolated by crossing single F1 DR-*white.mu* males that receive DSBs to *y w* females and molecularly analyzing the HR (y+ w+) F2 progeny. Of 41 HR repair events analyzed, the average gene conversion tract was 471.4 bp (±73.8). Six repair events (14.6%) were limited to the *Sac*I site, 17 (41.5%) were unidirectional, and the remaining 18 (43.9%) were bidirectional (Figure 4B). Of the unidirectional events, a majority (76.5%) converted to the right of the break, and the remaining tracts converted polymorphisms to the left of the break. Recovering both unidirectional and bidirectional events suggests that HR occurs from both one-ended invasion (unidirectional tracts) and two-ended invasion or Holliday junction migration (bidirectional; see *Discussion*).

HR repair is completed by either DSBR or SDSA, and these two models predict differences in the outcome of the donor sequence. hDNA formed during DSBR involves the donor sequence, which may subsequently be converted by mismatch repair, whereas the donor sequence remains unchanged in SDSA (Figure 1). The direct repeat feature in DR-*white.mu* allows analysis of the *iwhite.mu* donor sequence of all HR events for conversion of the polymorphisms. The *Sac*I site and the 13 closest polymorphisms (6 to the left, 7 to the right) were analyzed by amplification and sequencing the *iwhite.mu* donor sequence of the w+ isolates. Of the 41 HR repair events examined, none of the *iwhite.mu* polymorphisms were converted to the recipient *Sce.white* sequence (0/574 polymorphisms). Although gene conversion may involve the sister chromatid and is therefore lost in subsequent cell divisions (Johnson and Jasin 2000), it seems likely that because of the close proximity of the *white* repeat to the DSB, at least some portion of HR events would use the *white* repeat on the same chromatid. Additionally, DSBs that occur during G1 phase of the cell cycle would also require the repeat on the same chromatid as a template for HR repair in this system. As such, these data suggest that simple DSBs are repaired by an SDSA pathway, similarly to *P*-element excision gap repair (Adams *et al.* 2003; Kurkulos *et al.* 1994; Nassif *et al.* 1994).

### Recombination between diverged sequences is suppressed in *Drosophila*

**Figure 5:**
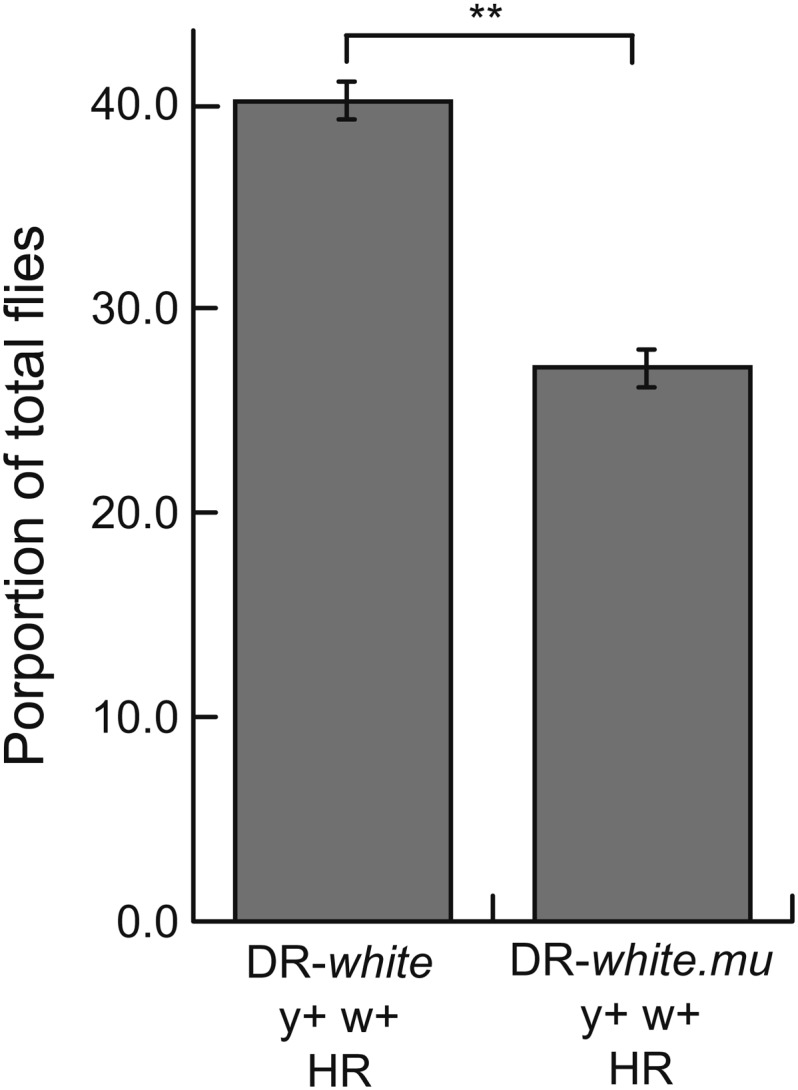
Recombination between diverged sequences is suppressed in *Drosophila*. Recombination between homologous (DR-*white*) and diverged (DR-*white.mu*) sequences was determined as in Figure 2. Average percentage with SD of HR events is shown from three independent experiments that simultaneously measured HR for both DR-*white* and DR-*white.mu*. DR-*white* recombination frequencies are from Figure 2. **P < 0.01 (pairwise Student *t* test). For total numbers of each experiment, see Table S2.

The additional silent polymorphism in DR-*white.mu* increases the sequence divergence between the direct repeats by 1.4%. This amount of sequence divergence strongly suppresses recombination in mouse embryonic stem cells and also suppresses recombination in human cells (Elliott and Jasin 2001; LaRocque and Jasin 2010). Because DR-*white* and DR-*white.mu* were targeted to the same locus in each line, recombination frequencies could be directly compared in wild-type DR-*white* and DR-*white.mu* flies to determine if recombination between diverged sequences is also suppressed in *Drosophila*. Similar to suppression in human cells, recombination between diverged sequences was suppressed by 31.5% (27.1 ± 1.2% HR with DR-*white.mu*) (Figure 5).

## Discussion

HR, SSA, and NHEJ repair of DSBs require both overlapping and unique factors, and these pathways result in varying intermediate and repair structures. Perhaps the most biologically significant difference among NHEJ, SSA, and HR is the molecular outcomes of these events. Whereas HR restores the genetic information at the site of the DSB, both NHEJ and SSA result in potentially mutagenic outcomes because of end processing and/or loss of genetic information at the site of the break. Classical NHEJ is often associated with 1–4 nucleotide insertions and deletions (Lieber *et al.* 2008). SSA requires a DSB between two direct repeats and involves annealing of resected repeats, resulting in loss of the intervening sequence (Ivanov *et al.* 1996). Pathway choice depends on the organism, cell-cycle phase, and cellular context of the lesion (Shrivastav *et al.* 2008).

Budding yeast are inefficient in imprecise NHEJ, which requires processing at the DSB ends, leading to changes in genetic sequences at the break. As such, DSBs are preferentially repaired by HR in yeast, particularly when in the diploid state (Aylon and Kupiec 2004). In higher eukaryotes, preference for HR or NHEJ is often regulated by cell-cycle phase, most likely because of availability of repair templates. HR is upregulated during S and G2 phase and NHEJ occurs throughout all cell-cycle phases, dominating G1 phase cells (Beucher *et al.* 2009; Delacote and Lopez 2008). In *Drosophila*, repair of a double-strand gap after *P*-element excision occurs through complete SDSA as frequently as aborted SDSA with end-joining (Adams *et al.* 2003; LaRocque *et al.* 2007), suggesting that repair initiated by strand invasion is a major repair pathway in this metazoan. Our results support this in that HR is the predominant repair pathway of all informative DSB repair events analyzed. Given that the y+ w− progeny may result from precise NHEJ, intersister HR, or no DSB, it is likely that the frequency of HR may be underestimated in this system. This may be especially true considering the large proportion of induced DSBs repaired by interhomolog recombination (Wei and Rong 2007).

Although our results demonstrate that HR is a dominant DSB repair pathway, the ability for the DR-*white* system to detect repair by HR, SSA, or NHEJ of a single DSB provides insight into DSB repair pathway choice. When the Rad51-dependent strand invasion step of HR is inhibited in *spn-A* mutants, we observe a significant increase in SSA rather than by NHEJ. The inability of *spn-A* mutants to strand invade resulted in extensive end resection of >7.2 kb, followed by SSA. This suggests that pathway choice occurs in several stages of the DSB repair process. Choice between HR/SSA and NHEJ is determined upstream of the end resection step in *Drosophila*; after initiation of processive end resection, NHEJ is no longer an option (Symington and Gautier 2011). Choice between HR and SSA is Rad51-dependent and occurs after end resection.

Previous studies demonstrate a preference for repair of a simple DSB by single-strand annealing, where an induced break occurs between two direct repeats that are in very close proximity to each other (Preston *et al.* 2006; Wei and Rong 2007). The location of these DSBs could potentially drive DSB repair by SSA, which requires end resection of only a few hundred base pairs and annealing of the repeats. Whereas SSA may be an important repair pathway when DSBs occur in repetitive DNA, these systems may be underestimating the contribution of HR when breaks occur in loci lacking direct repeats in close proximity. Additionally, Preston *et al.* (2006) utilized a constitutively active I-*Sce*I endonuclease. It is therefore difficult to delineate the contributions of DSB repair pathways, because constitutive breaking of the chromosome may lead to cell-cycle arrest and/or drive repair to a terminal repair event that eliminates the I-*Sce*I recognition sequence (Chan *et al.* 2011).

DR-*white.mu* allows for analysis of mitotic gene conversion tracts at high resolution. We found that the average gene conversion tract lengths in *Drosophila* were long (471.4 ± 73.8 bp) and were longer than gene conversion tracts in budding yeast (∼280 bp) (Cho *et al.* 1998) and in mouse embryonic stem cells (∼100 bp) (LaRocque and Jasin 2010). One possible explanation for the differences could be the length of homology available for recombination, because gene conversion tracts associated with unlimited homology availability during allelic recombination are long (Nickoloff *et al.* 1999). These systems do have differences in length of homology available for repair, because the longest homologous sequence is in the DR-*white* system (1.97 kb), followed by the reported yeast HR system (1.2 kb) and mammalian system (0.74 kb). However, even with adjusting for length of homology (average minimal tract length / total length of homology), gene conversion tract lengths in *Drosophila* are comparable to yeast (23.9% ± 3.7% and ∼23.25%, respectively), but longer than mammals (∼13.7%). Interestingly, during gap repair after *P*-element excision, 80% of aborted SDSA maintained repair synthesis of least 0.9 kb (Adams *et al.* 2003), and meiotic gene conversion tract lengths are also similar in *Drosophila* (Curtis *et al.* 1989; McMahan *et al.* 2013; Miller *et al.* 2012). Our results and those of others suggest that longer gene conversion tracts may be a general theme in *Drosophila*, in both mitotic cells and during meiotic recombination.

In addition to the longer length of gene conversion tracts, we found that 51.4% of gene conversion tracts extending beyond the *Sac*I polymorphism were bidirectional, similar to DSB repair of direct repeats in yeast (Palmer *et al.* 2003), but greater than in mouse cells (15%) (LaRocque and Jasin 2010). Directionality can be explained by SDSA associated with one-ended invasion, which would account for the unidirectional gene conversion tracts. The bidirectional tracts can be explained by Holliday junction formation followed by branch migration (Ferguson and Holloman 1996), gap repair (if short), or SDSA associated with two-ended invasion. However, analyses of the *iwhite* sequence demonstrated no changes to the donor sequence, which would be predicted at some frequency according to the DSBR model. Additionally, 92.3% of the exceptionally long gene conversion tracts (>600 bp) were bidirectional, and the average bidirectional tract length was significantly longer than unidirectional tracts (840.8 ± 106.9 bp for bidirectional, 246.3 ± 53.4 bp for unidirectional; *P* < 0.0001). Given that the donor sequence remained unchanged in our HR events, and that many bidirectional gene conversion tracts were very long, these data suggest repair by two-ended SDSA.

When divergence of the donor sequence increases by 1.4%, we observed a significant suppression of recombination. Sequence divergence of 0.5% does not affect meiotic recombination events (Hilliker *et al.* 1994), suggesting that either there is a threshold of divergence that exists for recombination suppression or, more likely, there is a conserved function to suppress aberrant recombination events between diverged sequences in mitotic cells, as observed in yeast and mammalian cells (Elliott and Jasin 2001; Nickoloff *et al.* 1999). Although this phenomenon is conserved, the extent of this suppression varies because mouse cells suppress recombination between sequences with 1.4% divergences to a greater extent than both human cells (LaRocque and Jasin 2010) and *Drosophila* (this study).

In conclusion, DR-*white* was established to demonstrate the large contribution of the *Drosophila* HR pathway in repair of a simple chromosomal DSB. Additionally, structures of gene conversion events were determined at high resolution using DR-*white.mu*. This work demonstrates how DSBs are repaired in wild-type organisms and supports the use of both DR-*white* and DR-*white.mu* to address various future questions in a genetically tractable whole organism, including (but not limited to) the role of genetic factors that are involved in DSB repair, effect of genomic context on DSB repair, repair in aged adult animals after an induced DSB, and DSB repair in the context of various life cycle stages and tissues.

## Supplementary Material

Supporting Information
