# Supplementary material for: Double-Strand Break Repair Assays Determine Pathway Choice and Structure of Gene Conversion Events in Drosophila melanogaster
Source: G3 (Bethesda). 2013 Dec 24;4(3):425–32. doi: 10.1534/g3.113.010074 (PMC3962482; doi:10.1534/g3.113.010074)
Supplement: Supporting Information [file supp_4_3_425__index.html]

Double-Strand Break Repair Assays Determine Pathway Choice and Structure of Gene Conversion Events in Drosophila melanogaster — Supporting Information 

# Double-Strand Break Repair Assays Determine Pathway Choice and Structure of Gene Conversion Events in *Drosophila melanogaster*

## Supporting Information for Do *et al.*, 2013

**Files in this Data Supplement:**

- Supporting Information - Tables S1 and S2 (PDF, 435 KB)
- Table S1 - Silent mutations (mu) in DR-*white.mu* (PDF, 425 KB)
- Table S2 - Total flies scored for each DSB repair phenotype (PDF, 64 KB)
