# Supplementary material for: Double-Strand Break Repair Assays Determine Pathway Choice and Structure of Gene Conversion Events in Drosophila melanogaster
Source: G3 (Bethesda). 2013 Dec 24;4(3):425–32. doi: 10.1534/g3.113.010074 (PMC3962482; doi:10.1534/g3.113.010074)
Supplement: Supporting Information [file supp_g3.113.010074_010074SI.pdf]

G3·Genes | Genomes | Genetics

Double-strand break repair assays determine pathway choice and structure of gene conversion events in *Drosophila melanogaster*

Anthony T. Do[*,1], Joseph T. Brooks[*,1], Margot K. Le Neveu[*], and Jeannine R. LaRocque[*]

[*]Department of Human Science, Georgetown University Medical Center, Washington, DC 20057

[1] These authors contributed equally to this work.

DOI: 10.1534/g3.113.010074

Table S1 Silent mutations (mu) in DR-*white.mu*

mu #	Distance (nt) from *Sac*I site[a]	nt change[b]	Restriction site change	mutagenic primer[c]
1	-864	C215A	*Pst*I+	5'-GCAAACACCCATCTGCAGAGCATCTGAACAATGG
2	-732	A347G	*Nhe*I+	5'-GTTCAGGGAGCGGCCAGCTAGCCGAGAACC
3	-693	C386T	*Eco*RV+	5'-CTGGCACAATATGGATATCTTTGGGGCGGTC
4	-645	C434T	*Hpa*I+	5'-GGCGGCAGCTGGTTAACCGGACACGCGGAC
5	-558	T521C	*Sma*I+	5'-TTTGCGGCGTGGCCTACCCGGGCGAACTTTTG
6	-516	A563C	*Nae*I+	5'-GTTCCGGTGCCGGCAAGACGACCCTGCTG
7	-471	C608G	*Bam*HI+	5'-TTTCGATCGCCGCAGGGGATCCAAGTATCG
8	-423	G656C	*Sal*I+	5'-GGCCAACCTGTCGACGCCAAGGAGATGC
9	-363	C716A	*Bam*HI+	5'-TGACCTCTTTATCGGATCCCTAACGGCCAG
10	-327	C752T	*Nco*I–	5'-GATTTTCCAGGCTATGGTGCGGATGCCACG
11	-276	G803T	*Bcl*I+	5'-GAGTGGCCCGCGTTGATCAGGTGATCCAGG
12	-201	T878C	*Stu*I+	5'-CAGGGTGAAAGGCCTGTCCGGCGGAGAAAG
13	-150	C929G	*Bam*HI+	5'-CCTCCGAGGCACTAACGGATCCGCCGCTTC
14	-63	G1016T	*Hind*III+	5'-GTGCTGAAGAAGCTTTCGCAGAAGGGCAAG
15	+32	A1110C	*Nci*I+	5'-TCTGATGGCCGAGGGCCGGGTAGCTTTC
16	+67	C1145G	*Sal*I–	5'-CCCAGCGAAGCCGTGGACTTCTTTTCCTAC
17	+118	G1196C	*Eag*I+	5'-CTACAATCCGGCCGACTTTTACGTACAGG
18	+190	A1268C	*Bgl*II+	5'-CGGATCGCCAAGATCTGCGACAATTTTGC
19	+236	T1314C	*Pvu*II+	5'-CCGGGATATGGAGCAGCTGTTGGCCACC
20	+304	C1382G	*Pml*I+	5'-CCTACAAGGCCACGTGGTTCATGCAGTTCC
21	+367	C1445T	*Xmn*I+	5'-CTCAAGGAACCACTTCTCGTAAAAGTGCG
22	+397	G1475C	*Nco*I+	5'-CTTATTCAGACAACCATGGTTGCCATCTTG
23	+472	A1550C	*Hae*II+	5'-TGAATATCAACGGCGCCATCTTCCTCTTCC
24	+547	G1625C	*Sac*I+	5'-TTCACCTCAGAGCTCCCAGTTTTTATGAGG
25	+613	G1691A	*Mfe*I+	5'-CTGGGCAAAACAATTGCCGAATTGCCGC
26	+694	A1772C	*Nae*I+	5'-GACTGCGGGCCGGCGTGCTGCACTTCTTC
27	+760	A1838G	*Eco*RV–	5'-GTGTCAACGTCCTTCGGGTATCTAATATCC
28	+859	C1937T	*Eco*RI+	5'-CGGCTTCTTCTTGAATTCGGGCTCGGTGCC

[a] Distance from I-*Sce*I site is determined by the location of the polymorphism relative to the wild-type *Sac*I cleavage site in *white* cDNA site used to create *Sce.white* (*Sac*I cleavage = 0, upstream of *Sac*I cleavage = –, downstream of *Sac*I cleavage = +).
[b] nucleotide change is determined by the nucleotide number of wild-type *white* mRNA sequence.
[c] forward primer given. Mutation is in red. Restriction site change is underlined.

Table S2 Total flies scored for each DSB repair phenotype

Genotype	DSB repair assay	Exp. no.	No. progeny scored (No. of individual germlines)	Total number of each phenotype (% of total)		
				y+ w– NHEJ, intersister HR, no DSB	y+ w+ intra-chromosomal HR	y– w– SSA
wild type	DR-*white*	1	1409 (18)	788 (55.9)	558 (39.6)	63 (4.47)
		2	891 (23)	509 (57.1)	349 (39.2)	33 (3.70)
		3	1659 (30)	929 (56.0)	699 (42.1)	31 (1.87)
		4	1726 (49)	1034 (59.9)	649 (37.6)	43 (2.49)
		**total**	**5685 (120)**	**3260 (57.3)**	**2255 (39.7)**	**170 (3.00)**
		**mean % (±S.E.M.)**		**57.2 (±0.93)**	**39.4 (±0.90)**	**3.13 (±0.59)**
	DR-*white.mu*	1	1441 (19)	1023 (71.0)	365 (25.3)	53 (3.68)
		2	602 (21)	416 (69.1)	162 (26.9)	24 (3.99)
		3	1226 (28)	850 (69.3)	362 (29.5)	14 (1.14)
		**total**	**3269 (68)**	**2289 (70.0)**	**889 (27.2)**	**91 (2.78)**
		**mean % (±S.E.M.)**		**69.8 (±0.60)**	**27.1 (±1.18)**	**2.9 (±0.91)**
spn-A/+	DR-*white*	1	710 (18)	398 (56.1)	293 (41.3)	19 (2.68)
		2	893 (20)	627 (70.2)	238 (26.7)	28 (3.14)
		3	401 (14)	246 (61.3)	139 (34.7)	16 (4.00)
		**total**	**2004 (52)**	**1271 (63.1)**	**670 (33.3)**	**63 (3.13)**
		**mean % (±S.E.M.)**		**62.5 (±4.13)**	**34.2 (±4.23)**	**3.35 (±0.46)**
spn-A/spn-A	DR-*white*	1	625 (17)	391 (62.6)	3 (0.48)	231 (37.0)
		2	1157 (27)	788 (68.1)	15 (1.30)	354 (30.6)
		3	631 (18)	401 (63.5)	5 (0.79)	225 (35.7)
		**total**	**2413 (62)**	**1580 (65.5)**	**23 (0.95)**	**810 (33.6)**
		**mean % (±S.E.M.)**		**64.7 (±1.71)**	**0.86 (±0.24)**	**34.4 (±1.94)**
