# Supplementary material for: Double-Strand Break Repair Assays Determine Pathway Choice and Structure of Gene Conversion Events in Drosophila melanogaster
Source: G3 (Bethesda). 2013 Dec 24;4(3):425–32. doi: 10.1534/g3.113.010074 (PMC3962482; doi:10.1534/g3.113.010074)
Supplement: Supporting Information [file supp_g3.113.010074_TableS1.pdf]

Table S1 Silent mutations (mu) in DR-*white.mu*

mu #	Distance (nt) from *Sac*I site[a]	nt change[b]	Restriction site change	mutagenic primer[c]
1	-864	C215A	*Pst*I+	5'-GCAAACACCCATCTGCAGAGCATCTGAACAATGG
2	-732	A347G	*Nhe*I+	5'-GTTCAGGGAGCGGCCAGCTAGCCGAGAACC
3	-693	C386T	*Eco*RV+	5'-CTGGCACAATATGGATATCTTTGGGGCGGTC
4	-645	C434T	*Hpa*I+	5'-GGCGGCAGCTGGTTAACCGGACACGCGGAC
5	-558	T521C	*Sma*I+	5'-TTTGCGGCGTGGCCTACCCGGGCGAACTTTTG
6	-516	A563C	*Nae*I+	5'-GTTCCGGTGCCGGCAAGACGACCCTGCTG
7	-471	C608G	*Bam*HI+	5'-TTTCGATCGCCGCAGGGGATCCAAGTATCG
8	-423	G656C	*Sal*I+	5'-GGCCAACCTGTCGACGCCAAGGAGATGC
9	-363	C716A	*Bam*HI+	5'-TGACCTCTTTATCGGATCCCTAACGGCCAG
10	-327	C752T	*Nco*I–	5'-GATTTTCCAGGCTATGGTGCGGATGCCACG
11	-276	G803T	*Bcl*I+	5'-GAGTGGCCCGCGTTGATCAGGTGATCCAGG
12	-201	T878C	*Stu*I+	5'-CAGGGTGAAAGGCCTGTCCGGCGGAGAAAG
13	-150	C929G	*Bam*HI+	5'-CCTCCGAGGCACTAACGGATCCGCCGCTTC
14	-63	G1016T	*Hind*III+	5'-GTGCTGAAGAAGCTTTCGCAGAAGGGCAAG
15	+32	A1110C	*Nci*I+	5'-TCTGATGGCCGAGGGCCGGGTAGCTTTC
16	+67	C1145G	*Sal*I–	5'-CCCAGCGAAGCCGTGGACTTCTTTTCCTAC
17	+118	G1196C	*Eag*I+	5'-CTACAATCCGGCCGACTTTTACGTACAGG
18	+190	A1268C	*Bgl*II+	5'-CGGATCGCCAAGATCTGCGACAATTTTGC
19	+236	T1314C	*Pvu*II+	5'-CCGGGATATGGAGCAGCTGTTGGCCACC
20	+304	C1382G	*Pml*I+	5'-CCTACAAGGCCACGTGGTTCATGCAGTTCC
21	+367	C1445T	*Xmn*I+	5'-CTCAAGGAACCACTTCTCGTAAAAGTGCG
22	+397	G1475C	*Nco*I+	5'-CTTATTCAGACAACCATGGTTGCCATCTTG
23	+472	A1550C	*Hae*II+	5'-TGAATATCAACGGCGCCATCTTCCTCTTCC
24	+547	G1625C	*Sac*I+	5'-TTCACCTCAGAGCTCCCAGTTTTTATGAGG
25	+613	G1691A	*Mfe*I+	5'-CTGGGCAAAACAATTGCCGAATTGCCGC
26	+694	A1772C	*Nae*I+	5'-GACTGCGGGCCGGCGTGCTGCACTTCTTC
27	+760	A1838G	*Eco*RV–	5'-GTGTCAACGTCCTTCGGGTATCTAATATCC
28	+859	C1937T	*Eco*RI+	5'-CGGCTTCTTCTTGAATTCGGGCTCGGTGCC

[a] Distance from I-*Sce*I site is determined by the location of the polymorphism relative to the wild-type *Sac*I cleavage site in *white* cDNA site used to create *Sce.white* (*Sac*I cleavage = 0, upstream of *Sac*I cleavage = –, downstream of *Sac*I cleavage = +).
[b] nucleotide change is determined by the nucleotide number of wild-type *white* mRNA sequence.
[c] forward primer given. Mutation is in red. Restriction site change is underlined.
