# Supplementary material for: Double-Strand Break Repair Assays Determine Pathway Choice and Structure of Gene Conversion Events in Drosophila melanogaster
Source: G3 (Bethesda). 2013 Dec 24;4(3):425–32. doi: 10.1534/g3.113.010074 (PMC3962482; doi:10.1534/g3.113.010074)
Supplement: Supporting Information [file supp_g3.113.010074_TableS2.pdf]

Table S2 Total flies scored for each DSB repair phenotype

Genotype	DSB repair assay	Exp. no.	No. progeny scored (No. of individual germlines)	Total number of each phenotype (% of total)		
				y+ w– NHEJ, intersister HR, no DSB	y+ w+ intra-chromosomal HR	y– w– SSA
wild type	DR-*white*	1	1409 (18)	788 (55.9)	558 (39.6)	63 (4.47)
		2	891 (23)	509 (57.1)	349 (39.2)	33 (3.70)
		3	1659 (30)	929 (56.0)	699 (42.1)	31 (1.87)
		4	1726 (49)	1034 (59.9)	649 (37.6)	43 (2.49)
		**total**	**5685 (120)**	**3260 (57.3)**	**2255 (39.7)**	**170 (3.00)**
		**mean % (±S.E.M.)**		**57.2 (±0.93)**	**39.4 (±0.90)**	**3.13 (±0.59)**
	DR-*white.mu*	1	1441 (19)	1023 (71.0)	365 (25.3)	53 (3.68)
		2	602 (21)	416 (69.1)	162 (26.9)	24 (3.99)
		3	1226 (28)	850 (69.3)	362 (29.5)	14 (1.14)
		**total**	**3269 (68)**	**2289 (70.0)**	**889 (27.2)**	**91 (2.78)**
		**mean % (±S.E.M.)**		**69.8 (±0.60)**	**27.1 (±1.18)**	**2.9 (±0.91)**
spn-A/+	DR-*white*	1	710 (18)	398 (56.1)	293 (41.3)	19 (2.68)
		2	893 (20)	627 (70.2)	238 (26.7)	28 (3.14)
		3	401 (14)	246 (61.3)	139 (34.7)	16 (4.00)
		**total**	**2004 (52)**	**1271 (63.1)**	**670 (33.3)**	**63 (3.13)**
		**mean % (±S.E.M.)**		**62.5 (±4.13)**	**34.2 (±4.23)**	**3.35 (±0.46)**
spn-A/spn-A	DR-*white*	1	625 (17)	391 (62.6)	3 (0.48)	231 (37.0)
		2	1157 (27)	788 (68.1)	15 (1.30)	354 (30.6)
		3	631 (18)	401 (63.5)	5 (0.79)	225 (35.7)
		**total**	**2413 (62)**	**1580 (65.5)**	**23 (0.95)**	**810 (33.6)**
		**mean % (±S.E.M.)**		**64.7 (±1.71)**	**0.86 (±0.24)**	**34.4 (±1.94)**
